# Targeted therapies and molecular targets in the therapeutic landscape of advanced urothelial carcinoma: state of the art and future perspectives

**DOI:** 10.37349/etat.2024.00279

**Published:** 2024-11-21

**Authors:** Irene Testi, Giulia Claire Giudice, Giuseppe Salfi, Martino Pedrani, Sara Merler, Fabio Turco, Luigi Tortola, Ursula Vogl

**Affiliations:** University of Bari, Italy; ^1^Oncology Institute of Southern Switzerland (IOSI), Ente Ospedaliero Cantonale (EOC), 6500 Bellinzona, Switzerland; ^2^Medical Oncology Unit, University Hospital of Parma, 43123 Parma, Italy; ^3^Institute of Oncology Research (IOR), 6500 Bellinzona, Switzerland; ^4^Department of Oncology and Hemato-Oncology, Università degli Studi di Milano, 20162 Milan, Italy; ^5^Section of Innovation Biomedicine-Oncology Area, Department of Engineering for Innovation Medicine, University of Verona and Verona University Hospital Trust, 37129 Verona, Italy; ^6^Faculty of Biomedical Sciences, Università della Svizzera Italiana, 6900 Lugano, Switzerland

**Keywords:** Advanced urothelial carcinoma, targeted therapy, bladder cancer, fibroblast growth factor receptor, TROP2, Nectin-4, TKIs, Her-2

## Abstract

Advanced urothelial carcinoma (aUC) has a dismal prognosis, with a 5-year survival rate of approximately 10%. Platinum-based chemotherapy has been the backbone of the first-line treatment of aUC for over 40 years. Only in the last decade, the treatment of aUC has evolved and been enriched with new classes of drugs that demonstrated pivotal improvements in terms of oncological responses and, ultimately, survival. Thus, the approach to aUC is becoming more and more tailored to the single patient, particularly owing to targeted therapies, such as fibroblast growth factor receptor (FGFR) inhibitors, antibody-drug conjugates (ADCs) targeting TROP2 and Nectin-4, anti-Her-2 therapies and others. However, due to the rapidly evolving scenario, the optimal sequence of systemic treatment is unknown and several important research questions remain unanswered, including the identification of reliable biomarkers to guide treatment decisions. Through ongoing research and clinical trials, we can continue to refine personalized treatment strategies and ultimately enhance patient care in this challenging disease setting. In this review, we provide a comprehensive overview of the current and emerging landscape of targeted therapies for aUC. We delved into the opportunities and challenges presented by personalized treatment approaches and explored potential future directions in this rapidly evolving field.

## Introduction

Urothelial carcinoma (UC) is the most common cancer of the urinary tract system, arising from the upper or the lower tract. Bladder cancer (BC) constitutes the majority (90%) of UC cases, accounting for 4.2% of all new cancer diagnoses worldwide [1]. Elderly patients are more frequently affected, with an average age at diagnosis of 73 years [1]. BC is classified into non-muscle-invasive BC (NMIBC), representing 75% of new diagnoses, and muscle-invasive BC (MIBC) [2]. While patients with NMIBC, invading up to lamina propria, have an excellent 5-year survival rate of 96%, the prognosis of advanced MIBC is dismal, with a 5-year survival rate of 8% [1]. The primary treatment for NMIBC is transurethral resection of bladder tumor (TURBT) followed by adjuvant intravesical therapy, either chemotherapy or Bacillus Calmette-Guérin (BCG) instillations, in case of high-risk features [3]. BCG-unresponsive or intolerant cases may require radical cystectomy [4] or therapy with pembrolizumab in regions where approved [Food and Drug Administration (FDA) only] [5]. Recently, nadofaragene firadenovec gene therapy has gained FDA approval for BCG-unresponsive high-risk NMIBC [6]. For localized MIBC in cisplatinum-eligible patients, the standard treatment involves neoadjuvant platinum-based chemotherapy followed by radical cystectomy [4]. Recently, an adjuvant treatment with nivolumab has been approved for high-risk resected disease [7]. Patients who are ineligible for surgery may undergo a trimodality treatment, with maximal TURBT, followed by concurrent chemoradiotherapy [4]. Unfortunately, around 10–15% of patients present with metastatic disease at diagnosis. Platinum-based chemotherapy has been the backbone of the first-line treatment for over 40 years, with the more recent introduction of maintenance avelumab in 2020 [8]. Nevertheless, the results of the up-to-date randomized phase III trials have completely revolutionized the treatment of advanced UC (aUC). The addition of nivolumab to the combination of cisplatin and gemcitabine showed a significant benefit in overall survival (OS) and progression-free survival (PFS) in the Checkmate 901 study, leading to recent FDA approval in first-line therapy [9]. The therapeutic revolution has come about through the identification of new treatment strategies aimed at specific targets. In this setting, antibody-drug conjugates (ADCs) have played the main role in the last few years’ improvements. Enfortumab vedotin (EV), a Nectin-4-binding antibody linked to a tubulin cytotoxin, previously approved in the third-line setting, was lately evaluated combined with pembrolizumab in treatment-naïve patients. At the European Society of Medical Oncology (ESMO) congress 2023, the clinical trial EV-302 received a standing ovation from the audience; following the outstanding results of this trial, the combination was recently FDA approved in first-line treatment [10], therefore, the treatment paradigm has recently changed dramatically with the exciting possibility of administering the combination in first-line therapy in clinical practice. Furthermore, sacituzumab govitecan (SG), a trophoblast cell surface antigen 2 (TROP2)-targeting ADC connected to a topoisomerase inhibitor, has been approved by the FDA for refractory UC disease [11]. Lastly, trastuzumab deruxtecan (T-DXd), a topoisomerase I inhibitor linked to an antibody directed against the human epidermal growth factor receptor-2 (Her-2), has found space in the later-lines setting of patients with UC harboring the Her-2 mutation or amplification [12]. Ultimately, also tyrosine kinase inhibitors (TKIs) are playing a key role in these diseases. Indeed about 20% of aUC diseases are characterized by fibroblast growth factor receptor (FGFR) mutations or fusions [13], and erdafitinib, a pan-FGFR inhibitor, has been approved for refractory patients with FGFR alterations [14]. Provided that, the National Comprehensive Cancer Network (NCCN) guidelines recommend molecular testing for FGFR at diagnosis [4]. Her-2 testing is ideally going to be part of the clinical practice too.

Unfortunately, despite all the recent progress, the prognosis of patients with aUC remains unfavorable and mostly incurable.

A broad investigation of the molecular and genomic patterns of UC has led to the development of more personalized approaches. In the present review we summarized the most explored pathways engaged in the development of UC, with their clinical implications. In addition, we are attempting to depict the possible future treatment strategies.

## Targeted therapies

### Anti-Nectin-4

The Nectin family of adhesion molecules is composed of four members (Nectin-1 to Nectin-4), whose extracellular domains participate in the formation and maintenance of cell junctions, modulating cell adhesion and crosstalk with the immune local environment [15]. Nectin-4 is highly expressed in UC samples, including more than 80% of BCs [16] and more than 60% of upper tract urothelial carcinoma (UTUC) [17]. Interestingly, its expression remains consistent even in non-urothelial histotypes of BC, albeit in a lower percentage of patients [18].

EV is the first approved ADC directed against Nectin-4. This therapeutic approach works by delivering the microtubule-disrupting agent monomethyl auristatin E (MMAE) directly into cancer cells upon binding to Nectin-4, ultimately triggering apoptosis [16].

EV was the first ADC to show promising results in patients pretreated with platinum-based chemotherapy and immune checkpoint inhibitors (ICIs). Initial encouraging safety and efficacy data came from the phase I EV-101 study [18]. This paved the way for the phase II EV-201 single-arm trial, which enrolled over 120 patients previously treated with platinum-based chemotherapy and ICIs. The study demonstrated a compelling objective response rate (ORR) of 44%, including complete responses (CRs) in 12% of patients [19]. Another cohort of the EV-201 study enrolled 91 patients who were cisplatin-ineligible andpreviously treated only with an anti-PD-1 (anti-programmed cell death protein-1) or anti-PD-L1 (anti-programmed cell death ligand-1) antibody, and treated them with EV. This group achieved an even higher ORR of 51%, with CRs observed in 22% of patients [20]. This led to FDA and European Medicines Agency (EMA) providing accelerated approval for EV in the treatment of locally-advanced or metastatic UC (mUC) patients who previously received a PD-1 or PD-L1 inhibitor and platinum-containing chemotherapy, as well as for cisplatin-ineligible patients who received one or more prior lines of therapy [21].

The rapid incorporation of EV into clinical practice can be attributed to the limited efficacy of prior standard-of-care third-line treatments for aUC, with single-agent chemotherapy offering a median PFS of less than 6 months. The phase III EV-301 trial directly compared EV to single-agent chemotherapy in 608 patients with progressive disease after prior platinum chemotherapy and ICIs. EV demonstrated a significant improvement in both PFS (5.55 months vs. 3.71 months) and OS (12.88 months vs. 8.97 months) while maintaining a similar safety profile [22]. While generally well-tolerated, the most common side effects associated with EV monotherapy include fatigue, hair loss, nausea, peripheral neuropathy, skin reactions, diarrhea, and hyperglycemia. These are typically mild to moderate in severity [23]. However, it’s crucial to acknowledge the potential for severe and even fatal cutaneous adverse events (AEs) like Stevens-Johnson syndrome and toxic epidermal necrolysis in some patients treated with EV.

However, significant advancements have emerged from the combination of EV with ICIs in UC treatment. A pivotal phase I/IIb EV-103 trial comprised a dose escalation/A cohort that included 45 patients who were platinum-ineligible and were treated with EV in association with pembrolizumab. Within this cohort, a promising ORR of 73.3% (CR in 15.6% of the patients) was shown. Furthermore, the median duration of response (DOR) reached 22.1 months, while median PFS and OS stood at 12.7 months and 26.1 months, over a 4-year follow-up period [24]. Building upon these promising results, within the same EV-103 study, investigators randomized 149 cisplatin-ineligible patients to receive either EV plus pembrolizumab vs. EV alone in the cohort K. Importantly, no statistical comparison was planned between the two treatment arms. The combined treatment arm displayed a noteworthy ORR of 64.5%, with median DOR, PFS, and OS yet to be reached after a median follow-up of 17.6 months [25]. Subgroup analyses reinforced these findings, demonstrating consistent ORR across patients irrespective of age, performance status, baseline metastasis sites (including liver metastasis), primary disease site of origin (upper tract vs bladder), and PD-L1 status [26]. As a result, FDA granted approval for the EV-pembrolizumab combination as a first-line therapeutic option for aUC patients ineligible for platinum-based chemotherapy.

Such favourable outcomes fostered the activation of a larger phase III randomized controlled trial (RCT), EV-302/KEYNOTE 39A, whose results have currently brought a major revolution to the standard of care first-line treatment for patients with aUC. EV-302 trial included 886 treatment-naïve patients with locally advanced or metastatic disease. Patients were randomized to receive either EV plus pembrolizumab or standard chemotherapic regimens (gemcitabine plus cisplatin or carboplatin, depending on eligibility to receive cisplatin). After a median follow-up of 17.2 months impressive breakthrough results were shown. Remarkably, patients treated with the EV-pembrolizumab combination exhibited significantly prolonged median PFS [12.5 months vs. 6.3 months; hazard ratio (HR) 0.45] and OS (31.5 months vs. 16.1 months; HR 0.47), compared to the chemotherapy-treated group [10]. The survival advantages noted in the intention-to-treat population were consistent through all the prespecified subgroups, including UTUC vs. lower tract, visceral or node only metastases, cisplatin elegibility and PD-L1 expression, both for OS and PFS [10]. Furthermore, at the ESMO 2024 congress, the impact of Nectin-4 expression was reported. Three subgroups were classified according to Nectin-4 expression and ORR, PFS and OS benefit were consistent in all subgroups. Additionally, Nectin-4 expression was not a predictive factor of EV-pembrolizumab combination response [27]. After several decades, for the first time, conventional platinum-based chemotherapy had been surpassed as the preferred first-line treatment for aUC and mUC. Based on these results, the combination of EV plus pembrolizumab garnered approval from both regulatory agencies FDA and EMA as the frontline treatment for adult patients with previously untreated locally advanced or mUC. Further exploration of survival data from subgroups of patients enrolled in the EV-302 trial, particularly those that received avelumab maintenance treatment and those that received third-line single agent EV, will allow to better integrate this new standard of care first-line treatment into the clinical practice and optimizing the treatment sequence upon disease progression [28].

The phase I Double Antibody Drug Conjugate (DAD) trial explored the combination of two ADCs, EV, and SG after two lines of therapy, with an ORR of 70% (95% CI, 47–87%) with 3/23 patients achieving CR and an encouraging safety profile, with the commonest grade ≥ 3 AEs (78% of patients) being neutropenia, anemia, and fatigue [29].

Additional cohorts within the EV-103 trial, alongside numerous ongoing trials, are currently exploring the efficacy of EV in combination with a variety of other drugs across various therapeutic contexts for aUC (Table 1).

**Table 1:** Selected ongoing clinical trials evaluating emerging targeted treatments in patients affected by advanced or metastatic urothelial carcinoma

**Clinicaltrials.gov registration number/name**	**Phase**	**Drug**	**Type of drug**	**Population**	**Setting**	**Number of patients**	**Endpoints**	**Current Status**	**Estimated primary completion date**
**Anti-Nectin 4**									
NCT03288545 EV-103 trial	Phase I/IIb randomized, multi-cohort, open-label, multicenter study	Dose escalation/cohort A, cohort B, and K: EV + pembrolizumab Cohort D: EV + cisplatin Cohort E: EV + carboplatin Cohort F: EV + gemcitabine Cohort E: EV + platinum + pembrolizumab Cohort H: EV	ADC, CT, ICI	Locally advanced or metastatic UC	First-line and/or refractory	348	ORR, DOR, DCR, PFS, EFS, OS, safety	Active, not recruiting	31-12-2026
NCT05923190	Phase II non-randomized two arm open-label de-esclation pilot study	EV ± pembrolizumab	ADC and ICI	Metastatic UC	First-line and/or refractory	70	OS, time to next treatment	Recruiting	01-07-2028
NCT05845814	Phase 1/2 randomized, umbrella study	Arm A: EV + favezelimab/pembrolizumab Arm B: EV + vibostolimab/pembrolizumab Arm C: EV + pembrolizumab	ADC and ICI	Locally advanced or metastatic UC	First-line	390	ORR, DOR, PFS, OS, safety	Active, not recruiting	31-05-2027
NCT03869190	Phase Ib/II, open-label, multicenter, randomized, umbrella study	2 arms: atezolizumab + EV	ADC and ICI	Locally advanced or metastatic UC	Refractory	645	ORR, DOR, DCR, PFS, EFS, OS	Enrollement is closed	06-12-2024
NCT04561362	Phase I/II, multicenter, first-in-human, open-label dose-escalation study	Zelenectide pevedotin alone and in combination with pembrolizumab	Bicycle toxin conjugate +/– ICI	Locally advanced or metastatic UC	Refractory, first-line standard-of-care-ineligible	329	ORR, PFS, OS, DOR, safety	Recruiting	12-2025
**Anti-TROP2**									
NCT04527991	Phase III, randomized, open-label study	SG vs. paclitaxel/docetaxel/vinflunine	ADC	Advanced or metastatic UC	Refractory	696	OS, PFS, ORR, CBR, DOR, safety, quality of life	Recruiting	20-2024
NCT03547973	Phase II open-label study	Cohort 3: SG + pembrolizumab Cohort 4: SG + cisplatin + avelumab/zimberelimab Cohort 5: SG + zimberelimab Cohort 6: SG +/– zimberelimab +/– domvanalimab	ADC and others	Advanced or metastatic UC	First-line and/or refractory	643	ORR, DOR, CBR, OS, PFS	Recruiting	07-2024
NCT04863885	Phase I/II non-randomized open label study	SG + nivolumab + ipilimumab	ADC and ICI	Metastatic urothelial bladder carcinoma	First-line, cisplatin-ineligible	46	ORR, DOR, PFS, OS	Active, not recruiting	11-10-2024
NCT03869190	Phase Ib/II, open-label, multicenter, randomized, umbrella study	1 arm: atzolizumab + SG	ADC and ICI	Locally advanced or metastatic UC	Refractory	645	ORR, DOR, DCR, PFS, EFS, OS	Closed enrollment	06-12-2024
NCT05327530	Phase II, multicenter, randomized, open label, parallel-arm, umbrella study	Arm B: avelumab + SG	ADC and TKI	Locally advanced or metastatic UC	Maintenance after first-line CT	252	ORR, DOR, PFS, OS, safety	Recruiting	23-01-2025
**FGFR inhibitors**									
NCT05544552	Phase I-II, multicenter, open-label	TYRA-300	FGFR 3-selective TKI	Advanced UC with activating FGFR3 gene alterations	Refractory	310	MTD, RP2D, ORR	Recruiting	11-2026
NCT03390504 THOR	Phase III	Erdafitinib	FGFR inhibitor	Advanced UC and selected FGFR gene aberrations	Refractory	629	OS, PFS, ORR, DOR, safety	Active, not recruiting	11-09-2024
NCT05775874	Phase II, single-arm，open-label, multicenter study	Fexagratinib + tislelizumab	FGFR inhibitor	Metastatic or locally advanced UC harboring FGFR alterations	Advanced	80	Safety, objective remission rate	Recruiting	30-09-2025
NCT02699606	Phase II, open-label, multicenter	Erdafitinib	FGFR inhibitor	Advanced non-small-cell lung cancer, UC, gastric cancer, esophageal cancer, or cholangiocarcinoma	Refractory	35	ORR, PFS, OS, DOR, safety	Completed	15-03-2024
NCT04601857	Phase II	Futibatinib + pembrolizumab	FGFR inhibitor and others	Advanced or metastatic UC	First-line	46	ORR, DCR, DOR, PFS, OS, safety	Active, not recruiting	05-2024
NCT03473756 FORT-2	Phase Ib/II	Rogaratinib + atezolizumab	FGFR inhibitor and anti-PD-L1	Advanced or metastatic UC	First-line	37	Safety, efficacy, RP2D, PK	Active, not recruiting	30-08-2024
**Anti Her-2**									
NCT02465060, NCT06136897 MATCH	Phase II, multicenter, non-randomized, open-label, multi-cohort	Genetic testing-directed monotherapies including: pertuzumab, trastuzumab, T-DM1, afatinib	MoAb, ADC	Her-2 amplified or mutated advanced unresectable or metastatic solid tumors, including UC	Refractory to standard treatment	6,452	ORR, OS, 6-months PFS	Active, not recruiting, has results arm B, J, Q	24-06-2024
NCT02122172	Phase II, multicenter, non-randomized, open-label	Afatinib	TKI	Her-2 amplified or mutated advanced unresectable or metastatic UC	Refractory to platinum +/– 1 other line	95	PFS, ORR	Active, recruiting	12-06-2024
NCT03602079	Phase I/II, multicenter, open-label	A166	ADC	Her-2 amplified or mutated metastatic solid tumors, including UC	Refractory	49	MTD, ORR, DLT, safety, Cmax	Completed	12-01-2022
NCT02675829	Phase II, multicenter, non-randomized, open-label	T-DM1	ADC	Her-2 amplified or mutated metastatic solid tumors, including UC	Advanced solid tumours	140	ORR	Active, recruiting	02-2025
NCT04482309 DESTINY-PanTumor02	Phase II, multicenter, non-randomized, open-label	T-DXd	ADC	Her-2 amplified or mutated advanced unresectable or metastatic solid tumors, including UC	Refractory	468	ORR, DOR, DCR, PFS, OS, safety	Active, not recruiting	30-07-2027
NCT04639219	Phase II, multicenter, non-randomized, non-label	T-DXd	ADC	Her-2 amplified or mutated advanced unresectable or metastatic solid tumors, including UC	Refractory	102	ORR, DOR, DCR, PFS, OS, safety	Active, not recruiting. Results posted	14-07-2026
NCT04839510	Phase II, multicenter, open-label	MRG002	ADC	Her-2 amplified or mutated advanced unresectable or metastatic UC	Refractory	58	ORR, DOR, TTR, DCR, PFS, OS, safety	NA	06-2022
NCT03809013	Phase II, multicenter, non-randomized open-label	DV	ADC	Her-2 amplified or mutated advanced unresectable or metastatic UC	Refractory	64	ORR, PFS, DOR, DCR, OS	Completed	05-06-2023
NCT04073602	Phase II, single center, non-randomized, open-label	DV	ADC	Her-2 amplified or mutated advanced unresectable or metastatic UC	Refractory	19	ORR, PFS, DOR, DCR, OS, safety	Completed	31-01-2023
NCT04319757	Phase Ib/II, multicenter, non-randomized, open-label	ACE 1702	NK cells	Her-2 amplified or mutated advanced unresectable or metastatic solid tumors, including UC	Refractory	36	DLT, safety	Active, recruiting	06-2024
NCT05318339	Phase II, multicenter, open-label	Trastuzumab + pyrotinib	MoAb + TKI	Her-2 amplified or mutated advanced unresectable or metastatic UC	Refractory	30	ORR, OS, PFS	Active, recruiting	10-12-2024
NCT04632992 MyTACTIC	Phase II, multicenter, non-randomized, open-label, multi-cohort	Cohort F and J: T-DM1 + atezolizumab Cohort G: trastuzumab + pertuzumab Cohort H: trastuzumab + pertuzumab + CT Cohort I: TDM1 + tucatinib	ADC + ICI, MoAbs, ADC + TKI	Her-2 amplified or mutated advanced unresectable or metastatic solid tumors, including UC	Refractory	252	ORR, PFS, DOR, OS, disease control, safety	Completed	27-02-2024
NCT04879329	Phase II, multicenter, open-label, multi-cohort	Cohort A, B, D: DV Cohort C and E: DV + pembrolizumab Cohort C randomized: DV + pembrolizumab vs. DV	ADC, ADC + ICI	Her-2 amplified or mutated advanced unresectable or metastatic UC	Cohort A, B, D: refractory Cohort C, E: I-line	332	ORR, safety, maximum and trough concentration, time to maximum concentration, DOR, PFS, DCR, OS	Active, recruiting	31-10-2024
NCT04644068 PETRA	Phase II, multicenter, open-label, multicohort	Module 1: AZD5305 Module 2: AZD5305 + paclitaxel Module 3: AZD5305 + carboplatin +/– paclitaxel Module 4: AZD5305 + T-DXd Module 5: AZD5305 + datopotamab-DXd Module 6: AZD5305 + camizestrant	PARPi, PARPi + CT, PARPi + ADC (including anti-Her-2)	Solid tumors, including UC	NA	804	Safety, DLT, best percentage change in target lesion, ORR, DOR, PFS, TTR, CA125 change, AUC, Cmax	Active, recruiting	15-12-2026
NCT05302284	Phase III, multicenter, randomized, open-label	DV + triplizumab vs. cisplatin/carboplatin + gemcitabine	ADC + ICI vs. CT	Her-2 amplified or mutated advanced unresectable or metastatic UC	First-line	452	OS, PFS, DOR, DCR	Active, recruiting	31-12-2026
NCT04278144	Phase I/II, multicenter, non-randomized, open-label	BDC-1001 +/– nivolumab	Immune-stimulating antibody conjugate +/– ICI	Her-2 amplified or mutated advanced unresectable or metastatic solid tumors, including UC	Refractory	390	Safety, MTD, DLT, ORR, DOR, DCR, PFS	Active, recruiting	31-01-2025
NCT04143711	Phase I/II, multicenter, non-randomized, open-label	DF1001	Trispecific antibody targeting Her-2, NK cells and T-cells	Her-2 amplified or mutated advanced unresectable or metastatic solid tumors, including UC (only in the escalation-expansion phases)	Refractory	378	DLT, ORR, safety, OS, DOR, PFS	Active, recruiting	10-2026
**PARP inhibitors**									
NCT03869190	Phase Ib/II, open-label, multicenter, randomized umbrella study	Niraparib	PARPi and others	Locally advanced or metastatic UC	Refractory	645	ORR, PFS, OS, DOR, DCR, safety	Recruiting	06-12-2024
NCT04678362 TALASUR	Phase II	Talazoparib + avelumab	PARPi and others	Platinum-sensitive locally advanced or metastatic UC	First-line maintenance treatment	50	PFS, OS, DOR	Recruiting	12-2023
NCT03375307	Phase II	Olaparib	PARPi	Metastatic or advanced UC and other genitourinary tumors with DNA-repair defects	Refractory	150	ORR, PFS, OS	Recruiting	21-08-2024
NCT03448718	Phase II	Olaparib	PARPi	Metastatic UC harboring somatic DNA damage response (DDR) alterations	Refractory	19	ORR, PFS, OS, safety	Completed Results posted	15-10-2021
NCT03682289	Phase II	Ceralasertib alone and in combination with olaparib or durvalumab	PARPi and others	Locally advanced or metastatic selected solid tumor malignancies	Refractory	89	ORR, DOR, PFS, safety	Recruiting	31-07-2025
**Multi-tyrosine-kinase inhibitors**									
NCT03425201 NICARAGUA	Phase I/II, multicenter, non-randomized, open-label	Cabozantinib + niraparib	Multi-TKI + PARPi	Advanced unresectable or metastatic UC or renal cell carcinoma	Refractory	20	MTD, PFS, satefy, ORR, DCR, DOR, OS	Active, not recruiting	04-2024
NCT03534804	Phase II, multicenter, non-randomized, open-label	Cabozantinib + pembrolizumab	Multi-TKI + ICI	Metastatic cisplatin-ineligible UC	First-line	34	ORR, 6-month PFS, OS, safety	Active, not recruiting	31-05-2024
NCT03866382	Phase II, non-randomized, open-label	Cabozantinib + nivolumab + ipilimumab	Multi-TKI + ICI	Metastatic rare genitourinary tumors	Refractory	224	ORR, PFS, OS, DCR, safety	Active, recruiting	28-02-2025
NCT05092958 MAIN-CAV	Phase III, multicenter, randomized, open-label	Cabozantinib + avelumab vs. avelumab	Multi-TKI + ICI vs. ICI	Advanced unresectable or metastatic UC	First-line maintenance treatment	654	OS, PFS, safety, tumor response, quality of life	Active, not recruiting	10-12-2024

ADC: antibody-drug conjugate; AUC: area under the curve; CAR: chimeric antigen receptor; CBR: clinical benefit rate; Cmax: maximum observed concentration; CT: chemotherapy; DCR: disease control rate; DLT: dose limiting toxicity; DOR: duration of response; DV: disitamab vedotin; EFS: event-free survival; EGFR: epidermal growth factor receptor; ICIs: immune checkpoint inhibitors; MTD: maximum tolerated dose; MoAb: monoclonal antibody; NA: not available; NK: natural killer; NMIBC: non-muscle-invasive bladder cancer; ORR: objective response rate; OS: overall survival; PARPi: poly(ADP-ribose) polymerase inhibitor; PFS: progression-free survival; RP2D: recommendend phase 2 dose; T-DXd: trastuzumab deruxtecan; T-DM1: trastuzumab emtansine; TKI: tyrosine kinase inhibitor; TTR: time to response; UC: urothelial carcinoma; UTUC: upper tract urothelial carcinoma; PD-L1: programmed cell death ligand-1; EV: enfortumab vedotin; FGFR: fibroblast growth factor receptor; Her-2: human epidermal growth factor receptor-2; PK: pharmacokinetics

Interestingly, it is worth mentioning that EV is not the only molecule targeting Nectin-4 that has been trialed in patients with UC.

Zelenectide pevedotin (BT8009) is a bicycle toxin conjugate (BTC), that acts by releasing MMAE into Nectin-4 positive cells, inducing cancer cell death [30]. BTCs are distinguished by their smaller size, which confers superior tumor penetration compared to ADCs, and have demonstrated lower rates of AEs in preliminary trials [31]. In the phase I/II BT8009-100 trial, 49 patients with advanced cancers expressing Nectin-4 were treated with BT8009, resulting in an ORR of 50% [32]. Several cohorts of the trial are ongoing, including those involving patients with UC, whether previously treated or untreated with EV.

In conclusion, targeting Nectin-4 represents a significant focus of current standard-of-care treatments for UC. Interestingly, a recent study by Klümper et al. [33] demonstrated that Nectin-4 amplification predicts EV response and long-term survival in patients with mUC, with a 96% response rate to EV therapy in amplified cases vs. 32% in others (*P* < 0.001). However, no selection is currently recommended for treatment indication. Further investigation into new combinations of EV across various settings, along with the introduction of additional Nectin-4 targeted therapies, holds promise for reshaping the treatment landscape of this disease.

### Anti-TROP2

**Figure 1:**
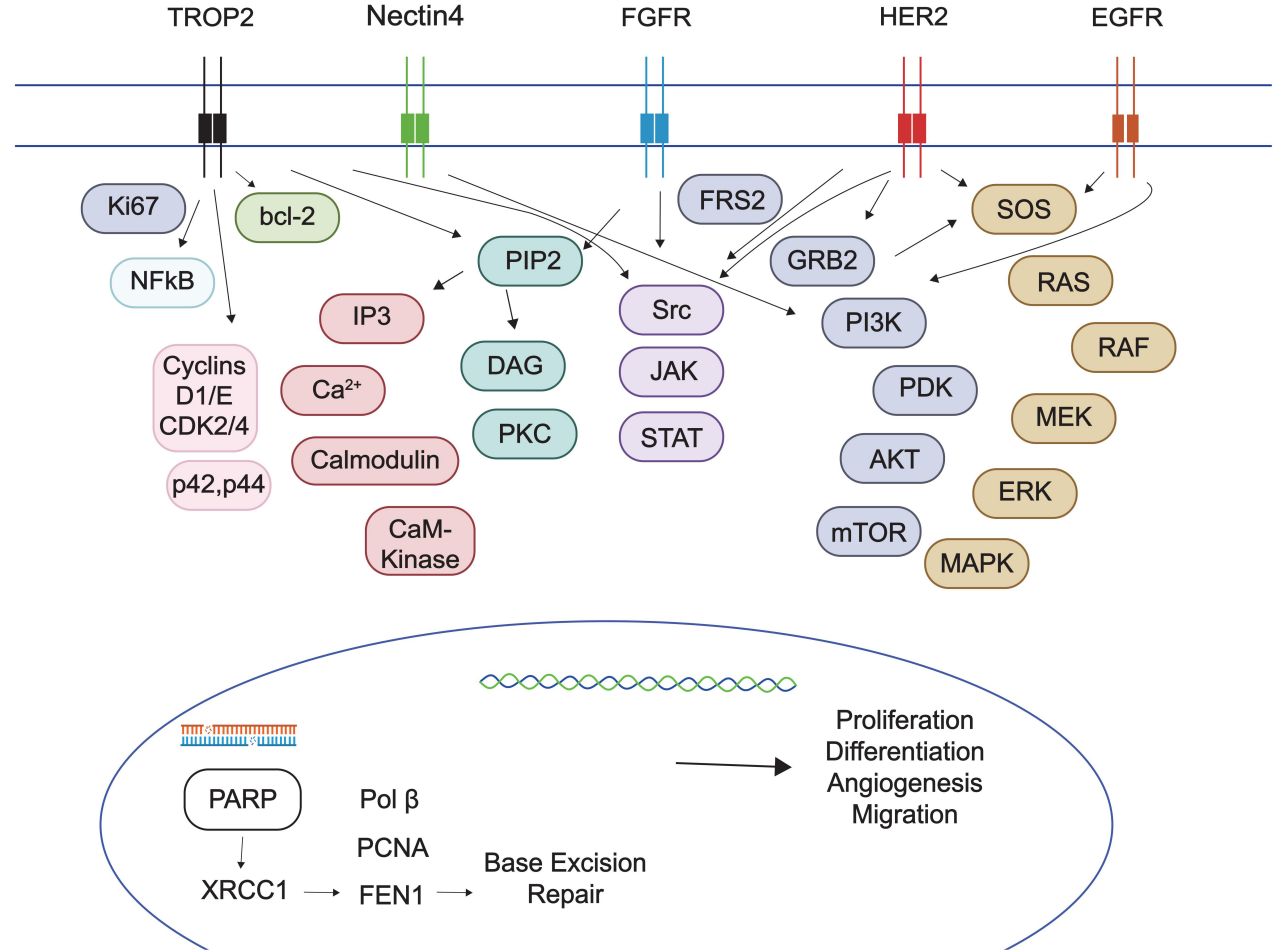
Multiple oncogenic pathways targeted in urothelial carcinoma. TROP2: trophoblast cell surface antigen 2; FGFR: fibroblast growth factor receptor; HER2: human epidermal growth factor receptor-2; EGFR: epidermal growth factor receptor; PARP: poly(ADP-ribose) polymerase

TROP2, encoded by *TACSTD2* gene, is a transmembrane glycoprotein involved in the activation of the ERK/mitogen-activated protein kinase (MAPK) pathway, promoting cancer cell proliferation, migration, invasion, and survival, also by regulating the calcium ion signaling pathway, cyclin expression and Ki67 expression [34, 35] (Figure 1). While highly expressed in normal urothelium [36], TROP2 is also overexpressed in various malignancies, including the majority of UCs of the bladder [37]. Its presence on the cell surface makes it an attractive target for antibody-based therapies.

SG represents the most developed drug targeting TROP2. It is a novel ADC consisting of a humanized anti-TROP2 monoclonal antibody coupled with the active metabolite of topoisomerase I inhibitor irinotecan, SN-38, via a hydrolysable linker [38].

The first efficacy data were collected in the IMMU-132-01 phase I/II basket trial, where SG was evaluated in advanced relapsed or refractory epithelial cancers [39]. Among the 45 patients with mUC enrolled in the trial, an ORR of 28.9% (13/45) was shown, fostering wider effort in trialing the drug in such disease context.

The TROPHY-U-01 (NCT03547973) phase II trial brought solid proofs of the efficacy and safety of SG in UC. This single-arm, multicohort study enrolled patients with locally advanced or metastatic disease. Patients were divided into various cohorts according to the previous treatment lines that they had received. Cohort I included 113 patients pre-treated with platinum-containing chemotherapy and either an anti-PD-1 or anti-PD-L1 antibody. The ORR for this group of patients was 28%; the median PFS was 5.4 months, and the median OS was 10.9 months [40]. Cohort II included 38 patients who were ineligible for a platinum-based first-line therapy and progressed after first-line ICI. In this cohort, ORR reached 32%, with a median PFS of 5.6 months [41]. Even better results were achieved in the third cohort, which enrolled 41 patients who were not previously treated with ICI and had disease progression after platinum-based chemotherapy in the metastatic setting or within 12 months of completion of platinum-based chemotherapy as neoadjuvant or adjuvant therapy. These patients were treated with SG plus pembrolizumab, showing a convincing 41% ORR, a median duration of treatment of 11.1 months, and a median PFS of 5.3 months [42]. Interestingly, TROP2 expression did not correlate with response, suggesting potential mechanisms beyond direct target engagement. Based on these findings, particularly from cohort I, SG received accelerated FDA approval for patients with locally advanced or mUC who have progressed after platinum-based chemotherapy and an ICI. Interestingly, ongoing cohorts 4 and 5 are investigating a combination of SG and cisplatin, with or without avelumab or zimberelimab (an anti-PD-1) induction therapy, followed by switch avelumab or zimberelimab maintenance.

SG was evaluated as safe, with the most frequently observed AEs being diarrhea (65%), nausea (60%), and fatigue (52%). These AEs led to SG discontinuation in 7% of patients, dose reduction in 40%, and dose interruption in 47%. AEs of grade ≥ 3 occurred in 65% of patients; the most common grade ≥ 3 TRAEs were neutropenia (35%), leukopenia (18%), and anemia (14%).

Multiple other ongoing clinical trials are employing SG in aUC. These include the multicentre phase III TROPiCs-04 trial, in which patients with locally advanced unresectable or mUC, progressing to platinum-based and ICIs are randomized to receive SG or physician’s choice single-agent chemotherapy [43]. Unfortunately, a recent press release announced the trial to be negative for OS [44].

Collectively, these data suggest that TROP2-targeted therapy with SG is emerging as a valuable strategy for a significant subpopulation of patients with mUC. Further research will refine our understanding of optimal treatment regimens and patient selection for this promising therapeutic approach.

### FGFR inhibitors

FGFR inhibitors occupy a central position in both the contemporary and prospective panorama of aUC [45]. Genetic modifications affecting the *FGFR* gene are detected in roughly 20% of cases of aUC and in approximately 36% of UTUC, potentially serving as oncogenic drivers [46].

FGFR signaling pathway assumes a pivotal role in different physiological cellular processes, including proliferation, survival, migration, differentiation, and angiogenesis, and it plays integral roles in embryogenesis, tissue regeneration and metabolic homeostasis [47, 48]. The FGFR family includes four transmembrane tyrosine kinase receptors, namely FGFR1, FGFR2, FGFR3, and FGFR4 [47]. The binding of an FGF ligand to an FGFR lead to the subsequent activation of multiple signal transduction pathways, such as RAS/MAPK (mitogen-activated protein kinase), phosphoinositide 3-kinase (PI3K; PI3K/Akt) and signal transducer and activator of transcription (STAT) [47] (Figure 1). Anomalies in FGFRs 1–4 encompass various aberrations, including fusions, mutations, amplifications, epigenetic or transcriptional dysregulation, or alterations within the tumor microenvironment (TME), culminating in the upregulation of FGF ligands [47, 48]. FGFR alterations represent viable targets for intervention through FGFR TKIs, as well as antibodies. FGFR TKIs can be categorized into FGFR1/2/3 inhibitors, FGFR4 inhibitors, pan-FGFR inhibitors, or multi-TKIs, the latter demonstrating activity against multiple protein tyrosine kinases, such as vascular endothelial growth factor receptor (VEGFR), platelet-derived growth factor receptor (PDGFR), and c-Kit [49]. Recent research strongly underscores the significance of FGFR inhibition in UTUC: an integrated analysis incorporating whole-exome sequencing and RNA sequencing of UTUC has unveiled that a majority of UTUC cases (approximately 60%) exhibit a molecular subtype (e.g., luminal-papillary subtype) characterized by elevated FGFR3 expression [50]. Furthermore, comprehensive genomic profiling of 2,463 UTUC and bladder UC cases has revealed a higher incidence of *FGFR3* gene alterations in UTUC compared to bladder UC (26% vs. 19%) [51].

Erdafitinib, a pan-FGFR1–4 inhibitor, achieved a milestone as the first targeted therapy FDA-approved in 2019 for previously treated FGFR mutated aUC patients. It has since emerged as the standard-of-care therapy for patients harboring *FGFR2/3* genetic alterations, particularly following prior treatment with platinum-based chemotherapy and ICIs [52].

In the phase 2, single-arm BLC2001 study involving patients with locally advanced or mUC bearing susceptible *FGFR2/3* alterations and progressing during or after chemotherapy, or within 12 months after neoadjuvant/adjuvant chemotherapy, and harboring one of nine prespecified *FGFR2/3* alterations (*FGFR3* mutations or *FGFR2/3* gene fusions), erdafitinib demonstrated significant efficacy [53]. With a follow-up duration of 24 months, 40% of patients who received erdafitinib achieved an objective response, including CRs in 3% and partial responses (PRs) in 37% of patients, with a median PFS of 5.5 months and a median OS of 13.8 months; the median time to response was 1.4 months, and the DOR was 5.6 months [54]. Grade ≥ 3 AEs occurred in 46% of patients, leading to discontinuation of treatment in 13% of subjects. Notably, the most common grade 3–4 AEs were stomatitis (14%) and hyponatremia (11%); any-grade hyperphosphataemia was seen in 77% of patients and grade 3 hyperphosphataemia in 2% of patients; other caveat include ocular disorders such as central serous retinopathy and retinal detachment [55].

The THOR trial is a confirmatory, phase 3, randomized study, involving patients with previously treated mUC, divided into two cohorts. In cohort 1, the objective was to evaluate whether erdafitinib could improve survival compared to chemotherapy among refractory patients with FGFR-altered mUC, after one or two previous treatments, including ICIs; in cohort 2, erdafitinib was compared with pembrolizumab in ICIs-naïve patients [14, 56]. Notably, salvage treatment with erdafitinib demonstrated superior survival outcomes compared to chemotherapy in cohort 1, with a median OS of 12.1 months vs. 7.8 months, respectively (HR 0.64; *P* = 0.005). The median PFS was also longer with erdafitinib compared to chemotherapy (5.6 months vs. 2.7 months; HR 0.58; 95% CI, 0.44 to 0.78; *P* < 0.001) and objective response was higher with erdafitinib (45.6% vs. 11.5%; relative benefit, 3.94; 95% CI, 2.37 to 6.57; *P* < 0.001). In the erdafitinib group, 6.6% of patients had a CR, and 39.0% had a PR; in the chemotherapy group, 0.8% had a CR, and 10.8% had a PR. The median DOR was 4.9 months (95% CI, 3.8 to 7.5) in the erdafitinib group and 5.6 months (95% CI, 2.1 to 6.0) in the chemotherapy group, with a similar incidence of grade 3 or 4 treatment-related AEs in the two groups (45.9% with erdafitinib vs. 46.4% with chemotherapy); treatment-related AEs that led to death were less common with erdafitinib (in 0.7% vs. 5.4% of patients, respectively). The most common treatment-related AEs of grade 3 or higher in erdafinitib group were palmar-plantar erythrodysesthesia syndrome (9.6%), stomatitis (8.1%), onycholysis (5.9%), and hyperphosphataemia (5.2%) [56]. On the other hand, erdafitinib did not show superior survival compared to pembrolizumab in cohort 2, with median survival of 10.9 months vs. 11.1 months, respectively (HR 1.18; *P* = 0.18) [14]. Interestingly, there were promising indications of greater benefit with erdafitinib among patients with UTUC, although this observation warrants cautious interpretation due to the underpowered nature of the sub-analysis [56].

In 2021, the phase II NORSE trial reported the outcomes of erdafitinib in combination with the anti-PD-1 antibody cetrelimab as first-line therapy for treatment-naïve patients with mUC who were ineligible for cisplatin-based chemotherapy. At a median follow-up duration of 14.2 months, the combinationdemonstrated an ORR of 54.5%, compared to 44% with erdafitinib monotherapy, with a CR rate of 13.6%. Also, median OS and PFS were longer with the combination (OS: 20.8 months vs. 16.2 months, PFS 10.9 months vs. 5.6 months) [57]. Although erdafitinib is currently the only FGFR inhibitor approved in mUC, several other molecules targeting FGFR have been investigated.

The first FGFR inhibitor examined in a clinical trial for UC was dovitinib, a multikinase inhibitor targeting various receptors including VEGFR, PDGFR, FGFR1-3 and c-KIT. Dovitinib displayed promising preclinical activity in bladder tumor cell lines and mouse xenografts characterized by *FGFR3* mutations, fusions, and overexpression. Preclinical investigations on UC cell lines revealed anti-proliferative effects and antiangiogenic properties [58]. Despite encouraging preclinical data, the drug yielded disappointing outcomes in a phase 2 trial [59]: dovitinib exhibited limited clinical efficacy irrespective of the underlying FGFR3 status in patients who had experienced disease progression following platinum-based chemotherapy, resulting in the premature termination of the trial.

Derazantinib, an oral multi-kinase inhibitor, exhibits activity against FGFR1–3, CSF1R, and VEGFR2. In a phase I study assessing its safety profile in patients with advanced or metastatic solid tumors, three patients reported a PR and, notably, among them there was a patient diagnosed with UC, characterized by *FGFR2* and FGF19 amplification [60]. Subsequently, the phase Ib/II clinical trial FIDES-02, which enrolled 303 patients with aUC harboring *FGFR* genetic aberrations, evaluated the ORR as well as the safety and tolerability of derazantinib administered as monotherapy or in combination with atezolizumab; the trial demonstrated modest clinical activity, with an ORR of 8%, a result considered insufficient to warrant further clinical development [61].

In preclinical xenograft models of BC, infigratinib, a selective FGFR1–3 inhibitor, demonstrated a reduction in tumor growth [62]. Following promising initial results from a phase I trial assessing infigratinib in patients with various advanced solid tumors harboring *FGFR* aberrations, including FGFR3-mutant UC [63], a subsequent evaluation in 67 pretreated patients with aUC characterized by *FGFR3* genetic alterations revealed an ORR of 25.4%. Notably, the ORR was approximately 31% in the early-line setting and 24% in the ≥ 2nd line setting, with 39% of patients exhibiting stable disease (SD) and a total disease control rate (DCR) of 64.2%, demonstrating significant activity, particularly in patients with UTUC [64]. Despite these promising clinical outcomes, AEs were reported in the majority of patients enrolled in the trial, with hyperphosphataemia (46.3%), elevated creatinine (41.8%), fatigue (37.3%), and constipation (37.3%) being the most commonly reported events of any grade. Commercial reasons led to the discontinuation of clinical testing of infigratinib.

In the phase 1 trial NCT01976741 the pan-FGFR1-4 TKI rogaratinib exhibited an ORR of 24% and a DCR of 73%. Notably, responses were observed in patients who had previously experienced disease progression on ICIs [65]. The phase II/III clinical trial FORT-1 (NCT03410693) was specifically established to make a direct comparison between rogaratinib and standard chemotherapy options (docetaxel, paclitaxel, or vinflunine) for patients with FGFR1/3 mRNA-positive locally advanced or mUC who had previously undergone treatment with platinum-based chemotherapy [66]. Among the 87 patients who received rogaratinib and the 88 patients treated with chemotherapy, the ORRs were 20.7% and 19.3%. Moreover, no significant differences were noted in OS (8.3 months vs. 9.8 months, *P* = 0.67), or PFS (2.7 months vs. 3.2 months, *P* = 0.86). However, a retrospective evaluation of patients exhibiting FGFR3 mRNA overexpression and FGFR3 DNA alterations showed ORRs of 52.4% for rogaratinib and 26.7% for chemotherapy, indicating a potential area for further research. Safety information from the trial indicated that grade 3 AEs occurred in 43% of the patients taking rogaratinib, compared to 39% in the chemotherapy group. Grade 4 events were noted in 4.7% and 18.3% of the two groups, respectively. The observed lack of effectiveness led to the halt of participant enrollment before advancing to the intended phase III segment of the study. The safety and effectiveness of first-line treatment using rogaratinib in combination with atezolizumab were investigated in cisplatin-ineligible patients with aUC exhibiting FGFR mRNA overexpression in the phase Ib/II FORT-2 study (NCT03473756) [67]. In this study, among 24 patients, 54% attained a confirmed objective response, including 3 CRs, leading to a DCR of 83%. The median DOR was not reached after a median follow-up of 7.4 months. Notably, a significant percentage (79%) of the 14 responding patients exhibited low or negative PD-L1 expression. Additional subgroup analysis of 16 patients with low or absent PD-L1 expression and no FGFR3 mutations or fusions demonstrated an ORR of 56%. This finding implies a promising activity of the combination of rogaratinib and atezolizumab, regardless of PD-L1 expression or FGFR3 mutation status. It is also important to note that the combination of an FGFR inhibitor with an ICI resulted in a higher occurrence of grade 3–4 AEs, impacting 54% of patients receiving the rogaratinib-atezolizumab combination.

Pemigatinib, an orally available FGFR1–3 TKI, is under investigation for its potential in UC. *In vitro* studies conducted on the BC cell line RT-4, which harbors the FGFR3-TACC3 translocation, demonstrated that pemigatinib markedly reduced FRS2 phosphorylation and activation of the MAPK signaling pathway. Additionally, *in vivo* studies on FGFR3-dependent models have shown that pemigatinib significantly suppresses tumor growth [68]. The safety profile of pemigatinib was evaluated in the FIGHT-101 trial, which included patients with various solid tumors, including UC. The most frequently reported AE was hyperphosphatemia (75%), while fatigue was the most common grade ≥ 3 AE (10.2%) [69]. In the single-arm phase II study FIGHT-201, pemigatinib was assessed in previously treated, unresectable, or mUC patients with FGFR3 alterations. Patients were divided into two cohorts: those with FGFR3 mutations or fusions/rearrangements (cohort A) and those with other FGF/FGFR alterations (cohort B). Patients received pemigatinib either continuously or intermittently. Among the enrolled patients, the ORRs were 17.8% and 23.3% for the continuous and intermittent dosing groups, respectively. The DORs were 6.2 months, and the median survivals were 6.8 months and 8.9 months, respectively. Similar ORRs of approximately 24% were observed among patients with the most common FGFR3 mutation (S249C) regardless of dosing schedule. However, pemigatinib demonstrated limited clinical activity in cohort B [70]. Pemigatinib was also investigated in the FIGHT-205 trial, a phase II randomized study, either as monotherapy or in combination with pembrolizumab as first-line therapy for cisplatin-ineligible patients with mUC. Unfortunately, the study was discontinued lacking interest to further develop the drug from the company [71].

Futibatinib (TAS-120), an irreversible inhibitor of FGFR1–4, has demonstrated promising antitumor activity in various cancer cell lines harboring FGFR alterations, including BC with FGFR3 fusions [72]. Futibatinib was evaluated in a large phase I dose-expansion trial comprising 197 patients with advanced solid tumors of different histologies [73]. Nineteen patients with UC were enrolled in the trial, achieving an ORR of 15.8%, including 3 PRs and 6 SDs, resulting in a DCR of 47.4%. These encouraging results were obtained in a heavily pretreated population, with over half of the enrolled patients having received more than three lines of prior treatment. Notably, 98.8% of enrolled patients experienced at least one AE. The most frequently reported AEs of any grade included hyperphosphatemia (81.2%), diarrhea (32.9%), constipation (31.8%), nausea (28.2%), fatigue (25.3%), and vomiting (25.3%). Grade 3 AEs were less common but included hyperphosphatemia (22.4%), increased alanine transaminase (9.4%), increased aspartate transaminase (5.3%), anemia (5.3%), and fatigue (5.3%). Currently, futibatinib is under investigation in BC patients harboring FGFR alterations in combination with pembrolizumab (see Table 1) [74].

Zoligratinib (Debio-1347), an oral selective inhibitor of FGFR1–3, in a phase I study conducted by Cleary et al. [75] was assessed in patients with advanced refractory solid tumors harboring FGFR genetic alterations. Notably, all enrolled patients experienced at least one AE, with the most common AEs reported in more than 25% of patients including hyperphosphatemia, diarrhea, nausea, fatigue, constipation, decreased appetite, nail changes, and dry mouth. Subsequently, the multicenter, open-label, phase II basket FUZE trial (NCT03834220) was initiated, aiming to enroll pretreated patients harboring FGFR-fusion positive tumors, regardless of histology, for treatment with zoligratinib. Unfortunately, the study was prematurely terminated in 2022 due to lower-than-expected antitumor activity [76].

Utilizing anti-FGFR monoclonal antibodies represents another avenue for targeting FGFR. Vofatamab is a fully human IgG1 monoclonal antibody specific for FGFR3. Preclinical studies have demonstrated its anti-tumor activity in xenograft models of BC and its anti-proliferative effect on FGFR3 cancer cell lines [77]. Vofatamab has been investigated in two different clinical trials: one evaluating its efficacy alone or in combination with chemotherapy (FIERCE-21, NCT02401542), and the other in combination with pembrolizumab (FIERCE-22, NCT03123055). Fierce-21 [78, 79] is a phase 1b/2 trial that investigated vofatamab as a standalone treatment or in combination with docetaxel for patients with mUC who were experiencing progression after platinum-based chemotherapy. The phase 2 expansion cohort of this trial included patients with FGFR3 mutations or fusions. Both the monotherapy and combination therapy groups demonstrated promising outcomes regarding DCR exceeding 25% in heavily pretreated patients. The treatment was well tolerated, with no reports of severe hyperphosphatemia or significant skin or ocular toxicities. In January 2019, vofatamab received fast-track designation from the FDA. The Fierce-22 study [80] assessed the effectiveness of combining vofatamab with pembrolizumab in platinum-refractory UC patients. In the monotherapy group, the ORR was 36% among all patients, 33% in the wild-type cohort, and 43% in the FGFR3 mutation/fusion cohort. Responses were observed at a median of 3.5 months, consistent with findings from the Fierce-21 study. As of 5 months, the median PFS had not yet been reached. Importantly, an analysis of paired biopsies taken before and after vofatamab monotherapy revealed that the treatment induced significant genomic alterations, notably an upregulation of genes linked to inflammatory responses and immune-gene modifications. These changes may support the rationale for advancing towards combinations with ICIs in patients who are molecularly unselected.

R3Mab (MFGR1877S) is a recombinant human antibody designed to selectively bind to the IgII and IgIII domains of FGFR3 shown to exhibit anti-tumor activity in *in vitro* studies, in BC models and in mouse xenograft models [81].

LY3076226 is an ADC comprising a fully human anti-FGFR3 antibody, IMC-D11, linked with a cleavable linker, sulfo-SPDB, to a cytotoxic tubulin inhibitor, maytansine derivative DM4. *In vitro* and *in vivo* studies have demonstrated its antitumor activity, resulting in cell cycle arrest, cell death, and tumor stasis, attributed to the cytotoxic payload of DM4 [82]. Although a phase I study (NCT02529553) evaluated the safety, efficacy, and pharmacokinetics of LY3076226 in patients with UC harboring FGFR3 alterations, the development of the drug was halted due to pipeline prioritization [83].

Finally, the novel small molecule FGFR inhibitor, fexagratinib, is currently undergoing clinical trials. In a phase II study involving tumors with aberrations of the FGFR pathway, fexagratinib demonstrated activity, with PRs observed in 8% of patients out of the 48% who were on therapy [84].

Furthermore, an ongoing phase II trial (NCT05775874) is investigating the safety and efficacy of fexagratinib in combination with tislelizumab, a humanized IgG4 anti-PD-1 inhibitor (Table 1).

### Anti-Her-2 targeted therapy

A further, profitable research field in the treatment of UC aimed at targeting Her-2, a tyrosine kinase membrane-bound receptor encoded by the *ERBB2* gene [85]. Her-2 is an epithelial growth factor receptor, involved in the signaling of cell proliferation, differentiation, and angiogenesis, through pathways such as the MAPK or the PI3K/Akt [86] (Figure 1). Her-2 overexpression, commonly found in breast, gastric, colon and lung cancer [86], is often caused by gene mutations (single base substitutions) or amplifications [87]. It is usually detected by immunohistochemical (IHC) staining and classified as negative (0/1+), equivocal (2+), and positive (3+). The equivocal samples are then confirmed with fluorescent in situ hybridization techniques, searching ERBB2 amplification [88]. Despite a lack of standardization in the Her-2 testing [89], its prevalence in UC samples ranges from 6.7% to 37.5%, according to a systematic review on 88 studies [90], without significant difference between early and aUC stages. In contrast, distinct evidence underlined a correlation with tumor grade and stage [91]; moreover, Her-2 prevalence appears to be raised in patients with luminal subtypes and UTUC [92, 93], and its amplification might lead to lymphatic dissemination [94] and poor prognosis in UC [95].

Several strategies of targeting Her-2 have been tested in patients affected by UC, either in monotherapy or in combination.

Trastuzumab is a monoclonal anti-Her-2 antibody, FDA and EMA approved for the treatment of breast and gastric cancer, as a monotherapy or in combination with chemotherapy. The activity as first-line therapy of trastuzumab in combination with chemotherapy (carboplatin, gemcitabine, and paclitaxel) was assessed in a phase II trial on 44 patients with aUC. The presence of Her-2 mutations or amplifications seemed to be related to a more aggressive disease with visceral metastases. The association exhibited an ORR of 70% with five CRs; the median PFS and OS were 9.3 months and 14.1 months, respectively. However, 75% of patients experienced grade 4 neutropenia and grade ≤ 3 cardiac toxicity, while two therapy-related deaths were reported [96]. Despite these results, in two following phase II trials, in both localized and advanced setting, the addition of trastuzumab to chemotherapy failed to prove superiority with respect to the standard treatment [97, 98].

In the multiple-basket trial MyPathway, the combination of trastuzumab and pertuzumab, a humanized monoclonal antibody directed against the extracellular domain of Her-2, was tested on a cohort of 346 patients affected by refractory solid tumors harboring mutations or amplifications of Her-2. Patients with UC (*n* = 32) showed an ORR of 21.1%, with two CRs. In addition, the coexistent mutation of KRAS was negatively associated with response to the combination therapy [99]. Table 1 summarizes the currently ongoing trials with anti-HER-2 monoclonal antibodies.

Several TKIs targeting Her-2 have been tested in UC, unfortunately without relevant results. TKIs generally act on the intracellular component of the receptor, interfering with its signaling.

Two phase II clinical trials assessed lapatinib on patients with pre-treated advanced solid tumors, including UC (*n* = 9 and *n* = 59, respectively), achieving DCR of 33% and 32% and ORRs of 0.00% and 0.01%, respectively [100, 101]. Additional attempts of combining lapatinib and chemotherapy did not increase benefit with respect to what expected, with ORR ranging from 8% to 59% and DCR from 39% to 82% [102, 103]. Ultimately, lapatinib was tested in a phase III trial, as a maintenance treatment on 232 patients affected by Her-1 or Her-2 mUC not progressed to a first-line platinum-based therapy; unfortunately, compared to placebo, the treatment did not add any benefit in terms of PFS or OS [104].

Afatinib, an irreversible inhibitor of epidermal growth factor receptor (EGFR), HER2, and HER4, initially showed modest DCR (21.7%) with a median PFS of 6.6 months, in a phase II trial on 23 patients with refractory aUC [105]; nevertheless, the benefit was not confirmed by subsequent trials [106].

Neratinib, a pan-Her TKI, demonstrated only a minor benefit in terms of DCR (18.8%) in a cohort of patients with UC, included in the basket SUMMIT trial [107]. Further evaluations on this TKI were not pursued on UC patients.

Lastly, preliminary evidence is available for pyrotinib, a novel irreversible pan-Her TKI, which was successfully administered to a patient with UC harboring Her-2 V842I mutation [108]. Ongoing trials are evaluating the activity and efficacy of TKIs in association with various therapies (Table 1).

As elucidated before, ADCs play an important role in mUC, and ADCs targeting Her-2 in UC are evolving.

Trastuzumab emtansine (T-DM1) is a Her-2 directed ADC, linked to an anti-microtubule agent; it was originally assessed in the phase II KAMELEON trial, enrolling patients with refractory solid tumors; unfortunately, the trial was held for scarce accrual. Nevertheless, in the thirteen patients affected by mUC, the treatment was safe and active, with an any-grade AE rate of 84.6% (grade 3 AE 30.8%) and an ORR of 38.5% [109].

Trastuzumab deruxtecan, a monoclonal antibody against Her-2 linked with a topoisomerase I inhibitor, has recently been approved by FDA for the treatment of pre-treated advanced Her-2-expressing (IHC 3+) neoplasms, based on the results of the DESTINY-PanTumor02, DESTINY-Lung02 and DESTINY-CRC01 trials [12, 110, 111]. The open-label phase II DESTINY-PanTumor02 trial evaluated T-DXd on 267 patients with pretreated solid tumors, including 41 mUC. The treatment was safe and active in this cohort of patients, with an ORR of 39%, a median PFS of 7 months, and a median OS of 12.8 months [12]. In addition, a combination therapy with T-DXd and nivolumab noted preliminary positive results in a phase Ib/II trial on patients with metastatic solid tumors, including 34 UC, who reported an ORR of 36.7%, a median PFS of 6.9 months and a median OS of 11 months [112].

An additional promising ADC is disitamab vedotin (DV), a novel anti-Her antibody, linked with a tubulin-disrupting antimitotic drug: MMAE. A pooled analysis of data from two phases II single-arm studies evaluating a total of 107 patients with mUC, who received DV in a treatment-refractory setting, reported an ORR of 50.5% and a median DOR, PFS, and OS of 7.3 months, 5.9 months, and 14.2 months, respectively [113]. The activity of DV was broadly evaluated in patients with mUC, in association with ICIs. The combination of DV with a PD-1 inhibitor (toripalimab or tislelizumab) was encouraging, showing an ORR of 88.9%, with five CRs and a median PFS of 12 months, in a real-world trial on nine patients [114]. A DCR and ORR of 87.5% and 62.5%, respectively, was seen in a cohort of sixteen pretreated patients [115] and an ORR of 83.3%, with a CR rate of 10%, in a phase Ib/II trial in 41 patients, 61% of whom treatment-naïve [116]. Moreover, given its apparent efficacy in patients with low Her-2 expression as well, a subsequent phase II trial aiming at evaluating the activity of DV on patients with Her-2 IHC 0 or 1+, was held. The ORR and the DCR of the nineteen patients were 26.3% and 94.7%, respectively [117].

Finally, trastuzumab duocarmazine, composed by trastuzumab and a DNA alkylator, has shown preliminary positive results; in a phase I trial held in patients with advanced solid tumors, the cohort of UC patients (*n* = 16) reached an ORR, a DCR and a median PFS of 25%, 94% and 4.0 months, respectively [118].

In general, the anti Her-2 drugs were well-tolerated; grade ≤ 3 nausea, vomiting and diarrhea were the most frequent AEs of TKIs, whereas ADCs treatment was burden by a low grade 3–4 AEs rate, mainly neutropenia and hepatic impairment. Specific ADC-related toxicities, such as hypoestesia for DV, ocular events for trastuzumab-duocarmazine and interstitial lung disease for T-DXd, were reported [12, 109, 113]. On the contrary, the combination strategy with ADCs and ICIs appeared to be less tolerated, with a grade 3–4 AEs rate of 73.5% with T-DXd plus nivolumab [112].

Further trials are evaluating several combination strategies of ADCs, with chemotherapy (trastuzumab-duocarmazine and paclitaxel), ICIs (T-DM1 plus atezolizumab or T-DXd plus nivolumab) or Her-2 TKIs (T-DM1 plus tucatinib), as well as novel ADCs, such as trastuzumab vedotin (MRG002). Ongoing studies are shown in Table 1.

### PARP inhibitors

Poly(ADP-ribose) polymerase (PARP) proteins play a crucial role in repairing genomic DNA damaged by free radicals or mutagens. Inhibition of PARP leads to the accumulation of DNA single-stranded breaks, ultimately promoting cell death. PARP inhibitors (PARPi) may exhibit enhanced efficacy in patients harboring mutations in the homologous recombination repair (HRR) pathway, such as BRCA1/2 alterations [119].

The prevalence of mutations in genes encoding proteins involved in the DNA damage response (DDR), such as BRCA1, BRCA2, ERCC2, and ATM, is up to 25% of patients diagnosed with aUC [13].

Despite the potential benefits, the use of PARPi is not currently standard practice for patients with UC, as none of the drugs in this class have received approval from regulatory agencies. Nonetheless, preliminary evidence suggests some activity of PARPi in UC treatment. Key questions remain unanswered, including which patients are most likely to benefit from PARPi, the optimal clinical setting for their use, and whether combination therapy with other agents, including ICIs, can enhance therapeutic outcomes [120, 121].

The PARPi rucaparib was investigated as salvage monotherapy for previously treated locally advanced or mUC in the ATLAS phase II trial, but it did not demonstrate significant activity regardless of homologous recombination deficiency (HRD) status. The study included 97 unselected patients with pre-treated aUC, of whom 20% had HRD-positive tumors, 31% had HRD-negative tumors, and 48% had indeterminate HRD status. There was no significant difference in median PFS between patients with HRD-positive or negative tumors (1.4 months vs. 1.8 months). No confirmed objective responses were observed in either the intention-to-treat (ITT) population or the subgroup of HRD patients [122, 123].

The efficacy of olaparib in previously treated aUC patients harboring germline BRCA1/2 mutations was demonstrated in a case report by Sweis et al. [123] in 2018. Building on this, a single-arm, open-label phase II study was conducted to evaluate the antitumor activity of olaparib in participants with mUC harboring somatic DDR alterations and who had progressed despite previous platinum-based chemotherapy or were cisplatin-ineligible [124]. Despite these genetic alterations, no patients achieved a PR to single-agent olaparib. However, six patients achieved SD, with durations ranging from 2.1 months to 16.1 months (median 7.69). The median PFS was 1.9 months, and the median OS was 9.5 months. These findings suggest that olaparib may have limited antitumor activity in patients with mUC and DDR alterations [124]. Two ongoing studies investigating the activity of olaparib in patients with advanced UC, NCT03375307 and NCT03448718, focus on patients with confirmed DDR alterations and have ORR as the primary endpoint (see Table 1).

The BAYOU phase II trial evaluated the addition of olaparib to durvalumab compared to durvalumab plus placebo in previously untreated platinum-ineligible patients with mUC, irrespective of HRR status [125]. Interestingly, no significant difference was observed between the two treatment arms in terms of PFS or OS in the ITT population. However, upon subgroup analysis, a statistically significant improvement in PFS was noted in patients with HRR mutations who received the combination therapy compared to those who received durvalumab alone (5.6 months vs. 1.8 months, HR 0.18, *P* < 0.001). This finding suggests that patients with HRR mutations may derive benefit from the addition of olaparib to durvalumab. The phase Ib BISCAY trial (NCT02546661) was designed as an adaptive, biomarker-directed study to evaluate the efficacy of durvalumab combined to various targeted therapies in platinum-resistant patients with [126]. The trial aimed to allocate patients to one of six treatment arms based on specific genomic alterations: single-agent fexagratinib or fexagratinib plus durvalumab in patients with FGFR3 alterations, olaparib in DDR genes alterations, vistusertib in patients with mutations of the PI3K-Akt-mTOR signaling pathway, durvalumab with or without olaparib in the event of none of the above-listed alterations (non-randomized control arm). Unfortunately, none of the treatment arms demonstrated encouraging ORRs that met the efficacy criteria for further development. Despite the lack of overall success, the combination of durvalumab and olaparib showed some promising results in certain patient populations. Specifically, among unselected patients receiving the durvalumab and olaparib combination, the ORR was 9.1%. In a subgroup of patients with *BRCA1/2*, *ATM* and *HRR* gene alterations (with approximately 50% of patients exhibiting high tumor mutational burden or positive PD-L1 expression), the ORR was 35.7%. This response rate was comparable to that observed in patients treated with durvalumab monotherapy (27.6%). Although the study was not designed to formally compare different treatments, the findings suggest that the combination of durvalumab and olaparib may be particularly effective in patients with specific genomic alterations associated with DNA repair deficiency. The investigators concluded that further evaluation of this combination strategy, particularly in platinum-naïve populations, is warranted.

The combination of niraparib plus cabozantinib in unselected treatment-refractory aUC patients is being investigated in the NICARAGUA trial (NCT03425201). This combination therapy aims to target multiple pathways implicated in tumor growth and survival, potentially enhancing treatment efficacy compared to single-agent therapies (see Table 1).

The phase Ib and II basket nonrandomized JAVELIN PARP Medley trial investigated the combination of talazoparib and avelumab in patients with advanced solid tumors, including 40 patients with UC who were not amenable to curative treatment. In this cohort, the ORR was 15.0%. Interestingly, the response rate was similar between patients who had received prior platinum therapy and those who had not (14.3% vs. 16.7%, respectively). Notably, one patient with a tumor harboring a BRCA alteration and negative PD-L1 expression achieved a CR, which was ongoing at the data cutoff point. These findings suggest that the combination of talazoparib and avelumab may hold promise as a treatment option for patients with aUC, particularly those with BRCA alterations [127].

Indeed, the TALASUR trial (NCT04678362) is exploring combination strategies for the treatment of aUC. By evaluating the efficacy of avelumab in combination with talazoparib as a maintenance therapy following platinum-based chemotherapy, this trial aims to provide valuable insights into the potential benefits of combining immunotherapy with PARPi in this patient population (Table 1) [128].

The MORPHEUS-UC trial (NCT03869190) is investigating the combination of atezolizumab and niraparib as second-line treatment for mUC following progression on platinum-based chemotherapy. The primary endpoint of this trial's mUC cohort is the ORR with the combination therapy, and definitive results are awaited (Table 1) [129].

The ATLANTIS trial marks a notable progression in the treatment of mUC by utilizing biomarker-driven allocation for switch maintenance targeted therapies. Patients who completed 4 cycles to 8 cycles of platinum-based chemotherapy without experiencing disease progression were eligible to participate in this adaptive, multi-comparison, randomized phase II platform trial. Patients were allocated to receive the PARPi rucaparib or matched placebo based on the presence of biomarkers associated with DDR alterations. Preliminary results from the ATLANTIS trial demonstrated that maintenance rucaparib extended median PFS compared to placebo, although this improvement did not reach statistical significance. While the difference in PFS was not statistically significant, the findings suggest that an adequate molecular selection strategy could be promising in the maintenance setting for patients with known DDR aberrations [130]. Further exploration in larger phase III trials is warranted to confirm the potential benefit of PARPi in selected patients with HRD-positive aUC.

Similarly, the Italian phase II Meet-URO-12 trial randomized 41 patients with aUC who had not experienced disease progression after platinum-based chemotherapy to receive maintenance niraparib alongside best supportive care (BSC) or BSC alone [131]. However, this study did not show a significant enhancement in median PFS with maintenance rucaparib, both in the overall patient population and among those with DDR alterations. The PFS was not markedly different between the two groups, with a median PFS of 2.1 months in the niraparib arm compared to 2.4 months in the control arm (HR 0.92, *P* = 0.81). The study’s enrollment was abruptly halted due to the availability of avelumab as a maintenance treatment in Italian clinical practice following platinum-based chemotherapy. Avelumab had demonstrated a significant improvement in OS compared to BSC, rendering further enrollment in the Meet-URO-12 trial unethical.

Overall, the results of ongoing trials, along with the potential conduct of randomized phase III studies, will be crucial in determining the optimal indications for PARPis in the treatment algorithm for patients with UC. Conducting further clinical trials in the described settings will help refine our understanding and utilization of PARPi in UC management.

### Other targeted therapies

Thus far, the potential involvement of numerous alternative signaling pathways has been investigated, albeit with limited success. Below we reviewed the most notable results.

The inhibition of the EGFR, with TKIs such as afatinib, lapatinib, or gefitinib, reported little efficacy [132–134]. To the best of our knowledge, only afatinib is currently being studied, in pre-treated patients with UC (see Table 1).

More interesting data emerged from a targeted inhibition of the VEGFR. In a neoadjuvant setting, the association of bevacizumab, a VEGF inhibitor monoclonal antibody, and chemotherapy showed a pathologic response rate (< pT2) of 53.0% [135], while two phase II trials on patients with aUC reported ORRs of 49% and 72%, with median OS of 13.9 months and 19.1 months, respectively [136, 137]. Unfortunately, these results were not subsequently confirmed in the phase III Alliance trial, not reporting any additional benefit of bevacizumab to a cisplatin and gemcitabine combination [138]. TKIs inhibiting multiple tyrosine-kinases, such as sorafenib and sunitinib, appeared to confer some benefit in ORR when added to chemotherapy, although further evaluations were not pursued due to high toxicity rates [139–143].

To date, cabozantinib, a mesenchymal epithelial transition (MET) and VEGFR inhibitor, emerged as the most promising TKI. When administered in monotherapy in a phase II trial on refractory UC patients, it proved an ORR of 19.5% and a median PFS and OS of 3.7 months and 8.1 months, respectively [144]. Moreover, based on the hypothesis of an immunomodulatory activity of cabozantinib, several trials evaluating its association with ICIs, in cohorts of pre-treated patients with UC, were held. Encouraging responses were noted, with ORR of 38.5% (in particular 16% in ICIs refractory patients) and 37.5% when combined to nivolumab (with or without ipilimumab) and durvalumab, respectively [145, 146]. Moreover, when added to atezolizumab in the COSMIC-021 trial, ORR of 30% and 27% were noted, when administered as a first-line or a second-line treatment, respectively [147, 148].

Similarly, the association of the multi-TKI, sitravatinib, to an ICI, nivolumab, showed a promising ORR of 31% [149], whereas lenvatinib failed to extend either OS or PFS when administered in combination with pembrolizumab in the phase III trial LEAP-011 [150].

Numerous trials evaluating a combination strategy with cabozantinib are ongoing (Table 1).

### Treatment choice and sequence

The past ten years have witnessed significant advancements in the treatment landscape for mUC, marked by the introduction of ICIs, ADCs, and targeted therapies. This expansion of therapeutic options has created a pressing need to determine the optimal sequencing and combination strategy to achieve the maximum clinical benefit while maintaining tolerability.

The goal of providing a unique international molecular definition of MIBC, ideally leading to a personalized treatment, was addressed by analyzing the transcriptomic profiles of 1,750 MIBC samples. In this study, six molecular subtypes were identified: luminal papillary (24%), luminal nonspecified (8%), luminal unstable (15%), stroma-rich (15%), basal/squamous (35%), and neuroendocrine-like (3%). Most significantly, these classes differ in underlying oncogenic mechanisms, in the frequency of target-genes mutations, in the immune and stromal infiltrate, as well as by histologic and clinical features, including long-term outcomes. In the future, the aim is to standardize and facilitate these stratifications, as well as to validate them in leading disease-tailored treatments. For example, luminal papillary tumors frequently present FGFR3 alterations, while basal ones present a higher rate of EGFR mutations and rich CD8 T cells and natural killer (NK) cells infiltrate [151]. Unfortunately, thus far, the molecular classification was not validated to guide treatment decisions.

**Figure 2:**
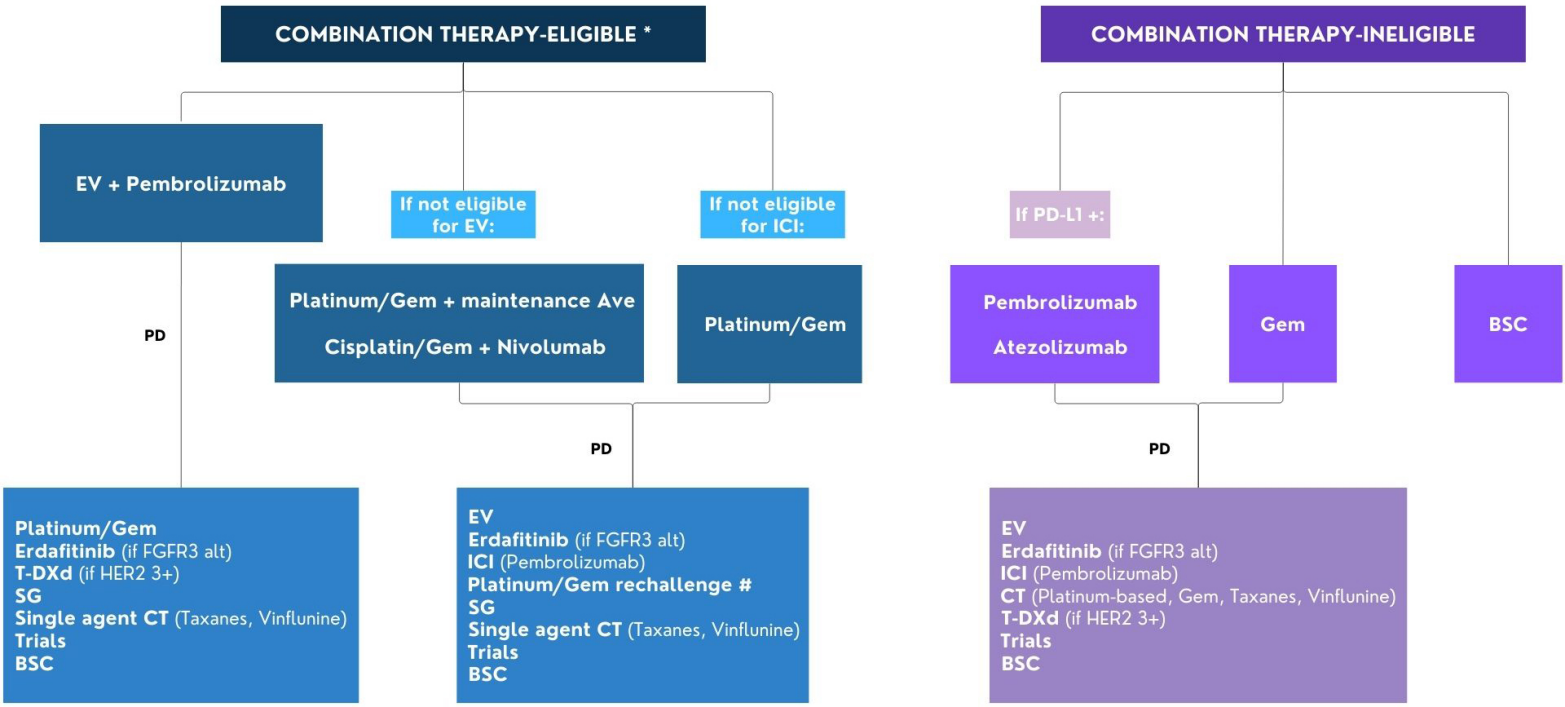
Flow chart for the management of patients with advanced/metastatic urothelial cancer. * Combination therapy eligibility: ECOG PS 0-2, GFR ≥ 30 ml/min, adequate organ functions; # Rechallenge with platinum/Gem if progression occurred ≥ 12 months after the end of previous platinum-based CT or ≥ 12 months after the end of previous platinum-based CT and maintenance avelumab. Alt: alterations; Ave: avelumab; BSC: best supportive care; CT: chemotherapy; EV: enfortumab vedotin; FGFR: fibroblast growth factor receptor; Gem: gemcitabine; ICI: immune checkpoint inhibitor; PD: progression disease; PD-L1: programmed cell death ligand-1; SG: sacituzumab govitecan; T-DXd: trastuzumab deruxtecan; ECOG PS: Eastern Cooperative Oncology Group Performance Status. This image was created using Canva, https://www.canva.com/

Consequently, several elements may be taken into account in clinical practice. Here, we depict the possible future treatment strategies with a proposal of how the various therapies can be used in combination or in sequence in the management of patients with advanced/metastatic UC (Figure 2).

For the past 20 years, chemotherapy has been the mainstay of treatment for mUC. However, the 2014 The Cancer Genome Atlas (TCGA) consensus marked the beginning of an era of personalized medicine for mUC [152]. In the wake of this consensus, several ICIs, including pembrolizumab and atezolizumab, gained approval, along with avelumab for maintenance therapy. The introduction of targeted therapies, particularly erdafitinib for patients with FGFR alterations, represented a significant shift in patient management. Additionally, the emergence of ADC, such as EV, has further transformed treatment paradigms.

Recently, international guidelines now recommend that most patients receive EV in combination with pembrolizumab as first-line therapy. For those ineligible for this combination or in regions where it is unavailable, the Galsky criteria should be utilized to determine cisplatin-eligible and cisplatin-ineligible patients. For cisplatin-eligible patients, the recommended regimen is cisplatin plus gemcitabine plus nivolumab, while for those ineligible, carboplatin plus gemcitabine is suggested, followed by maintenance with avelumab in non-progressive patients.

The incorporation of ICIs, such as pembrolizumab, atezolizumab, and nivolumab, has revolutionized the management of mUC, offering durable responses and improving survival outcomes for many patients [153–158]. Additionally, as described, ADCs like EV and SG have demonstrated efficacy in patients with relapsed or refractory disease; targeted therapies, especially FGFR and Her-2 inhibitors, have shown promising results in patients harboring specific alterations and mutations; PARPi have emerged as potential therapeutic options for patients with DNA damage repair deficiencies.

However, the abundance of treatment options presents challenges in determining the optimal sequencing and combination regimens. Factors such as tumor biology, prior treatments, patient comorbidities, and treatment goals must all be taken into account when making treatment decisions. Randomized clinical trials play a crucial role in these contexts to identify the most effective treatment strategies after ICIs failure and to elucidate the optimal sequencing or combination of therapies for patients with or without FGFR or Her-2 alterations (Table 1).

The optimal treatment strategy for aUC remains an area of active investigation. Tailoring treatment to individual patient needs is paramount, considering factors such as patient characteristics and disease features. Specifically, key considerations include ECOG performance status, kidney function, peripheral neuropathy, baseline cardiac risk, uncontrolled diabetes, ocular abnormalities andbiological age. Disease characteristics, including locally advanced or metastatic disease (such as lymph node-only disease vs. presence of visceral metastases), histology (pure vs. mixed), and site of origin (e.g., UTUC vs. BC), also play critical roles.

The incorporation of the EV-pembrolizumab combination as the preferred first-line systemic therapy represents a significant advancement in the treatment of mUC. This recommendation, endorsed by ESMO [159], EAU (European Association of Urology) [160] and NCCN guidelines [4], reflects the growing recognition of the efficacy and tolerability of this regimen.

In first-line treatment, the EV-302 trial evaluated both cisplatin-fit and cisplatin-unfit patients with locally advanced or mUC, yielding a mOS of 31.5 months compared to 16.1 months with standard chemotherapy (HR 0.47), with a median follow-up of 17.2 months [10]. To note, the EV-302 trial included even cisplatin-unfit patients, therefore, it showed a better survival in more fragile patients; indeed, in cisplatin-fit patients there is a huge advantage in terms of OS with the EV-pembrolizumab combination compared to the standard of care, but also in cisplatin-ineligible patients it seems to represent the best combination in terms of survival. In contrast, the Checkmate 901 trial focused exclusively on cisplatin-fit patients, reporting a mOS of 21.7 months vs. 18.9 months (HR 0.78), with a median follow-up of 33.6 months [9].

Finally, the Javelin Bladder 100 trial evidenced a mOS of 29.7 months vs. 20.5 months (HR 0.77), specifically a mOS of 31.5 months with cisplatin and 25.8 months with carboplatin, with a median follow-up ≥ 38 months [8]. In Javelin Bladder 100 the mOS is quiet similar to that observed in the EV-302 trial, although it is important to emphasize that the studies populations are different: in the EV-302 (and also in the Checkmate 901) study, all patients were treated in the first-line setting, whereas in the Javelin Bladder 100 study the population consists of patients who had not progressed within 3 months after the end of first-line therapy, therefore selected patients with better outcomes.

Subgroup analyses revealed important insights regarding specific populations. In the EV-302 trial, patients with liver metastases benefited significantly from the EV-pembrolizumab combination, achieving a mOS of 19.1 months compared to 10.1 months with chemotherapy; in Checkmate 901 trial this advantage is not clearly evident and in the Javelin Bladder 100 trial there is no mention of liver metastases, but rather of visceral metastases in general. Furthermore, the only trial to include Eastern Cooperative Oncology Group Performance Status (ECOG PS) 2 patients was EV-302, where both ECOG PS 1 and 2 patients demonstrated improved outcomes with the new combination; the Checkmate 901 trial only included ECOG PS 0 or 1 patients because, according to the Galsky criteria, it is not permissible to treat patients with ECOG PS 2 with cisplatin. Finally, in the Javelin Bladder 100 trial we have inclusion criteria for ECOG PS 2 and the advantage is more clear in patients with lower ECOG PS. Regarding kidney function, in the EV-302 trial (where the cutoff for creatinine clearance is ≥ 45 ml/min) there is a benefit with the new combination regardless of renal performance. Similarly, in the Javelin Bladder 100 trial (where the cutoff for creatinine clearance is ≤ 60 ml/min), there remains a benefit for patients who have received avelumab in the maintenance phase after prior chemotherapy. Lastly, with regard to the subgroup analysis of primary site tumor, this information is only mentioned in the subgroup analysis of the EV-302 trial, which shows no difference in outcomes based on the primary site; indeed, benefits are observed even in UTUC.

Regarding the safety profiles, it is crucial to select treatments balancing the benefit and the toxicity of therapies. The combination of EV and pembrolizumab is associated primarily with skin toxicity and peripheral neuropathy (AEs of any grade: skin toxicity 32.7%, peripheral neuropathy 50%, hyperglicemia 10.9%; AEs of grade ≥ 3: skin 7.7%, peripheral neuropathy 3.6%, hyperglicemia 5%, neutropenia 4.8%). Conversely, cisplatin plus gemcitabine plus nivolumab primarily incurs myelosuppression-related toxicities (AEs of any grade: skin toxicity 13.5%, neutropenia 30.6%, thrombocytopenia 21.7%, increased blood creatinine 12.8%; AEs of grade ≥ 3: skin 0.7%, anemia 22%, neutropenia 18%, increased blood creatinine 0.3%). During the avelumab maintenance phase of the Javelin Bladder 100 trial, the reported rates of AEs were as follows: skin toxicity 11.6%, fatigue 11.7%, diarrhea 16%, hypothyroidism 11.6%, and arthralgia 16.3%.

To note, quality of life (QoL) assessments across the three major first-line registration trials (EV-302, Javelin Bladder 100, Checkmate 901) reveal heterogeneous evaluation methods, with EV-302 showing improved QoL metrics compared to baseline. In contrast, in the Javelin Bladder 100 trial QoL is maintained during avelumab maintenance, while in the Checkmate 901 trial is indicated a slight decrease during the chemotherapy phase. Therefore, there is a huge need for standardized tools and assessment timelines to ensure consistency in clinical practice.

The sequence of treatments is critical, as high attrition rates in second-line therapies necessitate optimizing first-line options to maximize patient outcomes. With about only 37% of patients proceeding to second-line treatment after first-line failure, selecting the most effective first-line therapy is imperative [161, 162].

In conclusion, we advocate for EV plus pembrolizumab as the preferred first-line treatment for the majority of patients with aUC, relegating chemotherapy to second-line options. For patients where chemotherapy is deemed appropriate in the first-line, subsequent use of EV should be prioritized togheter with targeted therapies in presence of selective molecular alterations.

From our standpoint, the choice of first-line therapy with EV plus pembrolizumab should be considered in patients with high-burden disease scenarios, including visceral metastases, and ECOG PS 2. For cisplatin-ineligible patients, we recommend a flexible approach to treatment decisions that prioritizes patient well-being and potential benefits, rather than strictly adhering to trial criteria, such as creatinine clearance < 45 ml/min or pre-existing diabetes, because we do not know if patients with pre-existing comorbidities are at more risk to develop severe symptoms. There is ongoing discussion within the scientific community regarding eligibility criteria for the EV plus pembrolizumab combination. The EVITA criteria proposed by Prof. Grande et al. [163] suggest excluding patients at high risk for diabetes, neuropathy, ocular issues, and those with low kidney function or poor performance status. However, these criteria may unduly restrict access to potentially beneficial therapies.

In our opinion, the primary motivation for administering chemotherapy combined with nivolumab or avelumab, instead of EV-pembrolizumab, is the presence of lymph node-only disease. In such cases of low burden disease, we think we can minimize the toxicity associated with EV; indeed, in the post hoc analysis from Checkmate 901 trial presented at American Society of Clinical Oncology (ASCO) Genito-Urinary (GU) 2024, a 81.5% ORR and a 63% CR rate were observed in the subgroup of patients with lymph node-only disease treated with cisplatin/gemcitabine/nivolumab [164]. Similarly, in the Javelin Bladder 100 trial, patients with lymph node involvement responded well to platinum-gemcitabine combined with avelumab. These findings describe lymph node-only mUC as a distinct clinical entity and they reinforce the rationale for using nivolumab in conjunction with cisplatin-based chemotherapy as a standard first-line treatment option in this subgroup of mUC patients.

The changing of the first-line options is poised to reshape treatment selection strategies for mUC, potentially rendering many ongoing trials obsolete. With this paradigm shift, clinicians will need to navigate new challenges, including identifying the best management strategies for patients who show disease progression after first-line treatment with EV plus pembrolizumab. Whenever feasible, enrollment in clinical trials should be recommended. In the lack of data from randomized prospective clinical trials in this particular context, stratifying patients according to their FGFR and Her-2 mutation status may provide valuable insights for treatment decision-making. Understanding the molecular characteristics of patients who have received prior EV plus pembrolizumab could help identify potential candidates for subsequent therapies, such as erdafitinib, T-DXd, or other targeted agents.

The association between activating FGFR3 alterations and a T-cell-depleted phenotype in mUC raises important considerations for treatment decisions in these patients. While FGFR3 alterations may potentially confer resistance to ICIs due to the T-cell-depleted phenotype [50], the impact of these alterations on the response to ICIs remains uncertain. Studies suggest that the anti-tumor response to ICIs may not differ significantly between patients with and without FGFR3 alterations [45]. Given these considerations, the optimal treatment approach for patients with mUC and FGFR3 alterations remains unclear.

The findings from the TROPHY-U-01 cohort 1 study shed light on the potential interplay between different ADCs in the treatment of mUC. Despite some patients previously showing progression on EV, a significant proportion achieved a PR to SG. This suggests that, while there may be some degree of cross-resistance between these two agents, it is not universal, indicating the need for further investigation into their combined use. In this setting, the DAD trial demonstrated encouraging activity with the combination of EV and SG [29]. Conversely, the role of SG as second-line treatment, remains unclear, since a recent press release has announced the phase III trial negative for OS [44].

The retrospective studies highlighting the benefits of ICIs as the final treatment before EV, along with the correlation between cutaneous toxicities from EV and treatment response, provided valuable insights into the management of mUC. These findings emphasized the need for personalized treatment approaches, aimed at evaluating individual patient factors, treatment sequences, and AE profiles.

The improved ORR, median PFS, and OS observed in patients receiving ICIs before EV suggest a potential benefit of sequencing these therapies in a certain order. Understanding the impact of treatment sequence on outcomes can guide clinicians in making informed decisions regarding treatment selection and sequencing to optimize patient outcomes.

The debate surrounding the continuation of ICIs beyond disease progression or the modification of other systemic therapy is an important consideration in the management of mUC. While limited data are available on this concept, recent trials have shed some light on potential strategies to optimize treatment outcomes. In the phase II TITAN-TCC trial (NCT03219775), chemotherapy-refractory patients received nivolumab, followed by the addition of high-dose ipilimumab in case of early or late disease progression on nivolumab monotherapy. The addition of ipilimumab resulted in improved ORR of 33%, with a 7% of CRs. These findings suggest that adding ipilimumab to ongoing nivolumab therapy may offer value in certain patient populations, potentially enhancing treatment response [165]. Another multicohort trial explored the efficacy of an association of cabozantinib and nivolumab in ICIs-refractory mUC patients. Despite prior nonresponse to ICI monotherapy, the combination of cabozantinib and nivolumab demonstrated an ORR of 16%, with some patients experiencing responses. This observation suggests that cabozantinib may play a role in priming an effective immune response, even in patients who have failed prior ICI therapy [145]. These findings provide preliminary evidence supporting the concept of continuing ICIs alongside or after disease progression, either as monotherapy or in combination with other agents such as ipilimumab or cabozantinib. Further research, including larger prospective trials, is needed to confirm these findings and elucidate the optimal sequencing and combination strategies for maximizing treatment efficacy in patients with mUC.

Platinum rechallenge, although a well-established concept in the management of various malignancies, lacks prospective data supporting its use in patients with aUC [4]. However, retrospective studies suggest that platinum rechallenge could be advantageous for patients who are fit and still eligible for this treatment. Specifically, data from a substantial retrospective analysis suggest that disease control with platinum rechallenge is more probable in patients who previously achieved disease control with platinum-based chemotherapy, have a longer duration since their last platinum treatment, and/or have no detectable liver metastases [4]. Another potential strategy is platinum rechallenge following switch maintenance therapy with avelumab, although this approach also lacks prospective data to support its use [166].

Platinum-based chemotherapy may ultimately be opted on later lines. Finally, re-challenge with these new agents or ICI after ICI strategies, should only be considered inside clinical trials.

In the rapidly evolving landscape of treatment for mUC, ongoing studies are essential to determine the most effective therapeutic interventions following the failure of a EV plus pembrolizumab first-line treatment. However, interpreting the results of these studies may be challenging, especially those designed before the availability of EV, such as the THOR trial. Enhancements in trial design and execution are essential to tackle this challenge.

Researchers developing treatment strategies for patients with metastatic disease should take into account those who have previously been treated with ADCs and/or ICIs. It is crucial to address significant unmet needs, such as the creation of clinically relevant biomarkers. Therefore, future studies should focus on refining trial designs and incorporating robust biomarker analyses to improve outcomes for patients with aUC.

## Conclusions

Advanced UC represents a significant health challenge, with a 5-year survival rate of approximately 10%. Traditionally, platinum-based chemotherapy has been the mainstay of systemic management for aUC. However, the therapeutic landscape is rapidly changing due to the availability of various targeted therapies across multiple drug classes. FGFR inhibitors, anti-Her2 therapies, ADCs targeting TROP2 and Nectin-4, PARPi, and other TKIs are playing critical roles in the current and future treatment of aUC.

The expanding array of treatment choices for patients with aUC is leading to new clinical scenarios and complexities. As we navigate the challenges of advanced urothelial carcinoma treatment, there remain significant unmet clinical needs, including understanding treatment duration, dose management, toxicity mechanisms, and identifying predictive biomarkers for both efficacy and safety. Real-world data will be essential in refining our approach.

## Abbreviations

ADC: antibody-drug conjugate
AEs: adverse events
ASCO: American Society of Clinical Oncology
aUC: advanced urothelial carcinoma
BC: bladder cancer
BCG: Bacillus Calmette-Guérin
BSC: best supportive care
BTC: bicycle toxin conjugate
CR: complete response
DCR: disease control rate
DDR: DNA damage response
DOR: duration of response
DV: disitamab vedotin
EAU: European Association of Urology
ECOG PS: Eastern Cooperative Oncology Group Performance Status
EGFR: epidermal growth factor receptor
EMA: European Medicines Agency
ESMO: European Society of Medical Oncology
EV: enfortumab vedotin
FDA: Food and Drug Administration
FGFR: fibroblast growth factor receptor
GU: Genito-Urinary
Her-2: human epidermal growth factor receptor-2
HR: hazard ratio
HRD: homologous recombination deficiency
HRR: homologous recombination repair
ICI: immune checkpoint inhibitor
IHC: immunohistochemical
ITT: intention-to-treat
MAPK: mitogen-activated protein kinase
MET: mesenchymal epithelial transition
MIBC: muscle-invasive bladder cancer
MMAE: monomethyl auristatin E
mUC: metastatic urothelial carcinoma
NCCN: National Comprehensive Cancer Network
NK: natural killer
NMIBC: non-muscle-invasive bladder cancer
ORR: objective response rate
OS: overall survival
PARP: Poly(ADP-ribose) polymerase
PARPi: Poly(ADP-Ribose) Polymerase inhibitor
PD-1: programmed cell death protein-1
PD-L1: programmed cell death ligand-1
PFS: progression-free survival
PI3K: phosphoinositide 3-kinase
PK: pharmacokinetics
PRs: partial responses
QoL: quality of life
RCT: randomized controlled trial
SD: stable disease
SG: sacituzumab govitecan
STAT: signal transducer and activator of transcription
TCGA: The Cancer Genome Atlas
T-DM1: trastuzumab emtansine
T-DXd: trastuzumab deruxtecan
TKIs: tyrosine kinase inhibitors
TME: tumor microenvironment
TROP2: trophoblast cell surface antigen 2
TURBT: transurethral resection of bladder tumor
UC: urothelial carcinoma
UTUC: upper tract urothelial carcinoma
VEGFR: vascular endothelial growth factor receptor

## Declarations

### Copyright

© The Author(s) 2024.
